# Marine Actinobacteria: Screening for Predation Leads to the Discovery of Potential New Drugs against Multidrug-Resistant Bacteria

**DOI:** 10.3390/antibiotics9020091

**Published:** 2020-02-19

**Authors:** Manar Ibrahimi, Wassila Korichi, Mohamed Hafidi, Laurent Lemee, Yedir Ouhdouch, Souad Loqman

**Affiliations:** 1 Laboratory of Microbial Biotechnologies, Agrosciences and Environment (BioMAgE), Faculty of Sciences Semlalia, Cadi Ayyad University, PO Box 2390, Marrakesh, Morocco; wassila.korichi@edu.uca.ac.ma (W.K.); hafidi@uca.ma (M.H.); ouhdouch@uca.ac.ma (Y.O.); 2 Institut de Chimie des Milieux et Matériaux de Poitiers (IC2MP - CNRS UMR 7285), Université de Poitiers, 4 rue Michel Brunet – TSA 51106, 86073 Poitiers Cedex 9, France; laurent.lemee@univ-poitiers.fr; 3 Laboratory of Microbiology and Virology, Faculty of Medicine and Pharmacy, Cadi Ayyad University, PO Box 7010, Marrakesh, Morocco; s.loqman@uca.ma; 4 Agro Bio Sciences Program, Mohammed VI Polytechnic University (UM6P), Benguerir, 43150, Morocco

**Keywords:** marine habitats, isolation, screening, predator Actinobacteria, antibiotic, multidrug-resistant bacteria

## Abstract

Predatory bacteria constitute a heterogeneous group of prokaryotes able to lyse and feed on the cellular constituents of other bacteria in conditions of nutrient scarcity. In this study, we describe the isolation of Actinobacteria predator of other bacteria from the marine water of the Moroccan Atlantic coast. Only 4 Actinobacteria isolates showing strong predation capability against native or multidrug-resistant Gram-positive or Gram-negative bacteria were identified among 142 isolated potential predatory bacteria. These actinobacterial predators were shown to belong to the *Streptomyces* genus and to inhibit the growth of various native or multidrug-resistant micro-organisms, including *Micrococcus luteus*, *Staphylococcus aureus* (native and methicillin-resistant), and *Escherichia coli* (native and ampicillin-resistant). Even if no clear correlation could be established between the antibacterial activities of the selected predator Actinobacteria and their predatory activity, we cannot exclude that some specific bio-active secondary metabolites were produced in this context and contributed to the killing and lysis of the bacteria. Indeed, the co-cultivation of Actinobacteria with other bacteria is known to lead to the production of compounds that are not produced in monoculture. Furthermore, the production of specific antibiotics is linked to the composition of the growth media that, in our co-culture conditions, exclusively consisted of the components of the prey living cells. Interestingly, our strategy led to the isolation of bacteria with interesting inhibitory activity against methicillin-resistant *S. aureus* (MRSA) as well as against Gram-negative bacteria.

## 1. Introduction

In the late 1960s, the discovery of penicillin as the first antibiotic led to a significant reduction of mortality and infectious diseases caused by bacteria [1,2]. However, the discovery of new classes of antibiotics was limited in the late 1960s [3], and bacteria rapidly developed a varied array of antibiotic resistance mechanisms [4,5]. Consequently, the prevalence of multidrug-resistant (MDR) bacteria became a growing problem worldwide [6]. According to 2016–2017 WHO’s new Global Antimicrobial Surveillance System (GLASS) report, antibiotic resistance is a serious health threat worldwide.

Nowadays, very few antibiotics are active against multidrug-resistant bacteria [7] and classical pathogenic Gram-negative bacteria. To face this major global healthcare threat and thus combat and stop the spreading of antibiotic-resistant pathogens, it is imperative to discover new and efficient antibiotics with novel mechanisms of action [8,9]. Most of the known natural antibiotics, active against pathogenic microorganisms, are produced by Actinobacteria [10,11]. Actinobacteria could reduce the growth (bacteriostatic activity) or kill (bactericidal activity) other microorganisms such as bacteria, fungi, and even other Actinobacteria thanks to the production of secondary metabolites [12]. These bacteria are thus the most efficient producers of most marketed antibiotics [13,14]. Recent whole-genome sequencing programs have revealed that the biosynthetic potential of Actinobacteria has been greatly under-explored and thus under-exploited [15,16]. Cryptic gene clusters in Actinobacteria are now regarded as an untapped source of bacterial secondary metabolites [17]. Consequently, nowadays, various approaches are being tested to stimulate the production of microbial secondary metabolites by Actinobacteria [13,18,19,20,21].

Co-cultivation is one of these strategies [22,23,24] and has led to the isolation of 33 new secondary metabolites from 12 Actinobacteria [22].

The exploration of the biosynthetic abilities of predatory bacteria is another approach used to discover novel secondary metabolites [25,26]. Predatory bacteria constitute a heterogeneous group of prokaryotes that share the ability to feed on the cellular constituents of other bacteria [27]. They include *Ensifer adhaerens* [28], *Cupriavidus necator* [29,30], *Lysobacter spp.* [31,32], *Bdellovibrionales* (d-BALOs) [26,27,28], Myxobacteria [20,29,30], and Actinobacteria such as *Streptomyces atrovirens* [33]. The predatory activity of these bacteria involves the secretion of small bioactive molecules acting as predatory weapons and of lytic enzymes [34,35]. Chemical investigations of these bacteria led to the discovery of many new natural products with antibacterial activity, including gulmirecins [36], myxopyronins [37,38], corallopyronins [39], and althiomycin [26,40]. These secondary metabolites are thought to contribute to the killing of prey organisms, since in their absence, the predatory performance of these bacteria is severely affected [25,26]. The ecology and physiology of predator Actinobacteria has been poorly studied. Only a few *Streptomyces* species were reported to manifest predation behavior [41,42]. This might be due to the fact that they are facultative predators, so their predatory activity might not be detected under standard culture conditions. Indeed, predation is likely to be triggered in specific conditions such as reduced nutrients availability [42,43]. It would be interesting to determine whether the Actinobacteria cryptic secondary metabolites biosynthetic pathways are activated in the course of the predation process.

Banning (2010) [44] reported that predation could be extremely beneficial for bacteria living in oligotrophic marine environments, and marine Actinobacteria were shown to be highly metabolically active [45,46]. Morocco, because of its specific geographic position, bears a unique marine environment characterized by important temperature variations throughout the year [47]. These are influenced by the connection between the Gulf Stream, via the Azores Current and the Canary Current, and the North Equatorial Current [48]. Since the antagonistic potential of Actinobacteria from Moroccan marine environment has been poorly explored, we screened for new non-obligate predatory Actinobacteria from Moroccan marine water able to feed on other bacteria cellular constituents in conditions of nutritional stress. Furthermore, we demonstrated the production of secondary metabolites by these bacteria, but the putative role of these molecules in the predation process could not be firmly demonstrated.

## 2. Results

### 2.1. Physico-Chemical Parameters 

The sampling sites and the various physicochemical properties of the collected marine water are provided in Table S1. The sampling sites were characterized by rather high salinity (ranging from 35.1 mg/L to 35.8 mg/L) and conductivity (ranging from 53.4 mS/cm to 54.2 mS/cm). The pH of the water varied between 7.76 and 8.08.

### 2.2. Isolation and Screening of Predatory Actinobacteria 

**Figure 1:**
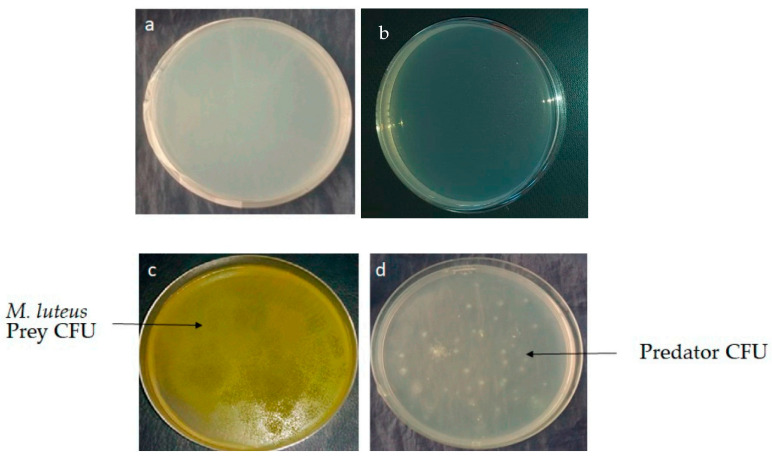
Predator isolation procedures and controls. (**a**) Plating of 1 mL of *Micrococcus luteus* at the concentration 10^12^ colony-forming units (CFU)/mL embedded in 20 mL of molten agar-agar at 45 °C: no growth detected; (**b**) plating of 100 µL of a water marine sample dilution on the surface of 20 mL of agar-agar: no growth detected; (**c**) growth of 1 mL of *M. luteus* at the concentration 10^12^ CFU/mL embedded in 20 mL of molten agar-agar at 45 °C flooded by 1 mL of nutrient broth; (**d**) growth of potential predators able to use *M. luteus* as a nutritional source.

In total, 142 morphologically different predatory bacteria were isolated from 9 water marine samples collected from different locations of the Atlantic Ocean along the Moroccan coast. After 14 to 21 days of incubation at 28 °C, predatory bacteria colony-forming units (CFU) appeared on agar-agar that contained just 1 mL of live washed cells of *Micrococcus luteus* at the concentration 10^12^ CFU/mL as the sole nutrient source (Figure 1d). The water samples were diluted up to 10^−6^ before plating. The isolation of predatory Actinobacteria was performed by three different methods described in Materials and Methods. *M. luteus* was chosen as the prey because it is the only reported prey for *Streptomyces* species with predation behavior, and the yellow color of its CFU facilitates its experimental monitoring. The appearance of predatorial CFU from marine water samples on agar-agar containing the prey indicated the presence of at least one predator (Figure 1d), whereas, in controls samples, no CFU were observed (Figure 1a,b). A control sample consisting of agar-agar flooded with nutrient broth confirmed the viability of the prey, as shown in Figure 1c.

The obtained CFU were purified in the same isolation medium to obtain pure cultures of the predatory bacteria after 72 to 96 h of incubation at 28 °C. These cultures showed filamentous morphology characteristics of Actinobacteria under light microscopy (G X10) as well as specific features described by the International *Streptomyces* Project (ISP) [49]. After purification, the isolates were stored in 20% sterile glycerol and frozen at −80 °C. These results confirmed that *M. luteus* can be used as a nutritional source by predatory Actinobacteria.

In total, morphologically different predatory bacteria were isolated from marine water samples originating from nine different locations of the Atlantic Ocean along the Moroccan coast.

### 2.3. Selection of Actinobacteria with Predatory Ability and Assessement of the Putative Prey Specificity of the Selected Actinobacteria Predators

After this primary screening, 142 bacteria isolates were obtained as potential predators. To assess their predatory ability, we tested each pure isolate individually for its aptitude to grow on agar-agar with live prey cells as the sole source of nutrients. Out of the all isolates, only four different morphotypes of predatory Actinobacteria, designed EMM111, EMM112, EMM183, and EMM194 were selected (Table 1). The selection was based on the following criteria: growth after 72–96 h of incubation, consistency, pigmentation of substrate and aerial mycelium, microscopic observation, and specific features defined by the ISP.

**Figure 2:**
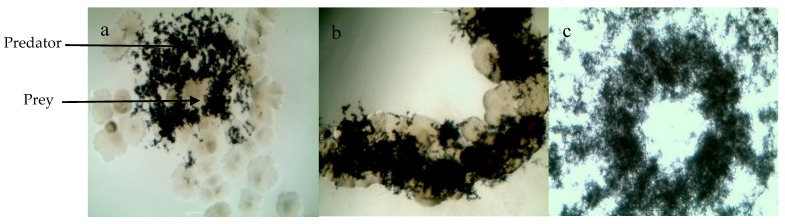
Images of Actinobacteria EMM111 predation of *M. luteus*. (**a**) and (**b**) Predation of *M. luteus* by Actinobacteria observed by phase-contrast microscopy at magnitude of 400x; (**c**) total elimination of prey cells after 15 days of incubation. The scale bar is 10 μm.

In order to determine the possible prey specificity of the four selected isolates, the latter were grown on agar-agar inoculated with the following Gram-positive and Gram-negative bacteria belonging to different taxonomic groups, *M. luteus* (ML), *Staphylococcus aureus* (SA), methicillin-resistant *S. aureus* (MRSA), *Escherichia coli* (EC), and ampicillin-resistant *E. coli* (AREC). The four strains tested showed clear macroscopic expanding zones on agar-agar on all preys (Table 2 and Figure 2). This indicated the total lack of specificity of this predation process.

### 2.4. Impact of Actinobacteria on M. Luteus Viability 

**Figure 3:**
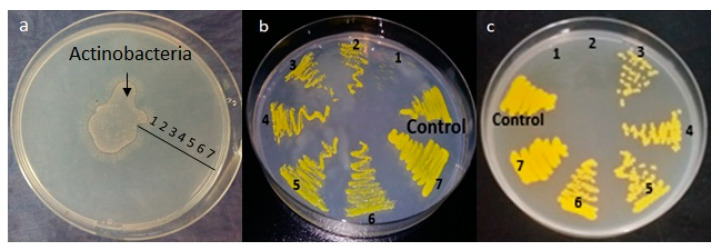
Impact of Actinobacteria on *M. luteus* viability. Pure culture growth of predatory Actinobacteria in the presence of washed live cells of *M. luteus* as the sole source of nutrients on agar-agar medium (**a**). Transplanted viable *M. luteus* prey cells present at various distances from Actinobacteria after 24 h (**b**) and 48 h (**c**) of incubation.

In an attempt to determine whether the predatory process involves the production of diffusible killing substances by Actinobacteria, the latter was deposited in the center of a plate of agar-agar containing *M. luteus* as a prey. Clearing zones signaling *M. luteus* lysis were clearly seen around the Actinobacteria deposit. *M. luteus* present at various distances (indicated with numbers from 1, i.e., proximal to 6, i.e., distal) from the Actinobacteria clearing zones was transplanted on nutrient agar (NA). After 24 h (Figure 3b) or 48 h (Figure 3c) of incubation, it was obvious that the growth of *M. luteus* originating from zones 1 and 2 was either absent or not as dense as that of *M. luteus* originating from zones 5 or 6 or from the control’s zone 7 incubated separately in the absence of predator (Figure 3b). This indicated that Actinobacteria had a clear negative impact on *M. luteus* viability, but we could not determine whether this was due to the predatory activity of Actinobacteria or to its ability to produce bactericidal substances.

### 2.5. Production of Antimicrobial Compounds by Actinobacteria Grown on Bennett Medium

**Figure 4:**
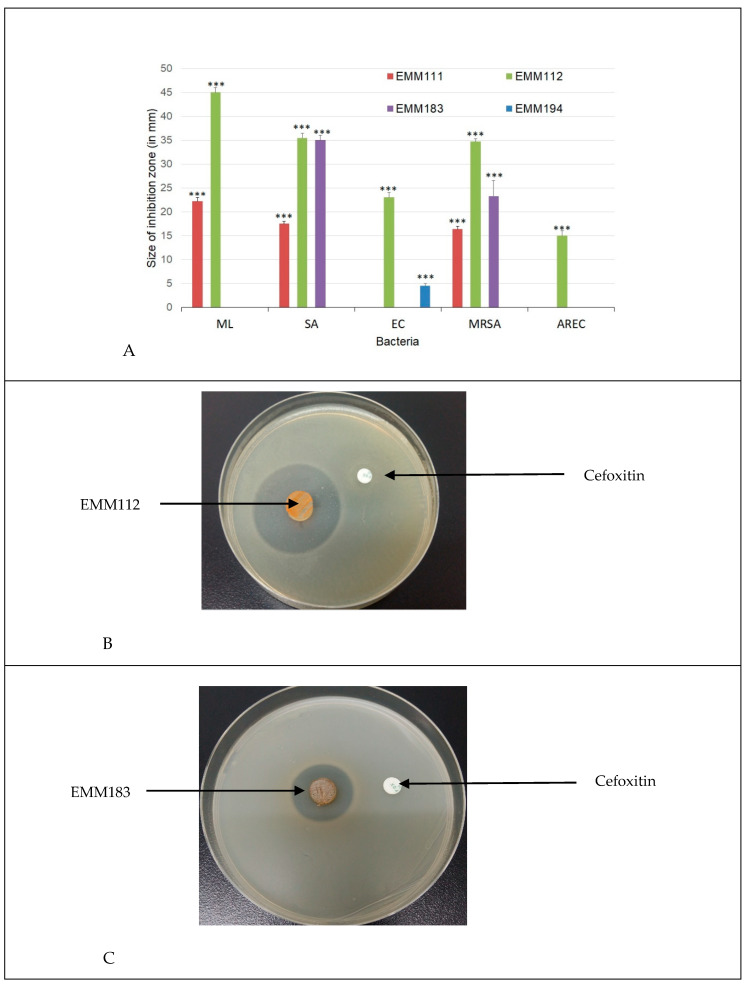
(**A**): Comparative bar graph showing the size of inhibition zones in mm for the four non-obligate predatory Actinobacteria using Bennett medium against different bacteria. Each value represents the mean ± SD of three replicates; ***p < 0.001 indicates significant differences compared the agar cylinders of Bennett medium using two-way analysis of variance (ANOVA). ML: *M. luteus,* S: *Staphylococcus aureus,* EC: *Escherichia coli*, MRSA: methicillin-resistant *S. aureus*, AREC: ampicillin-resistant *E. coli*; (**B**): Antibacterial activity of EMM112 using Bennett medium against MRSA; (**C**): Antibacterial activity of EMM183 using Bennett medium against MRSA.

Since the clear negative impact that Actinobacteria had a on *M. luteus* viability could be due to the production of antimicrobial substances, the production of antibacterial substances by the four predatory Actinobacteria isolates was assessed using Bennett medium (Table 2). Two Actinobacteria (EMM 111 and 112) produced substances limiting the growth of *M. luteus*, but EMM183 and 194 did not. Otherwise, most of the Actinobacteria showed a significant degree of antagonistic activities against all tested microorganisms (Figure 4A–C). Among the selected isolates, EMM111 had significant inhibitory activity against Gram-positive SA, MRSA, and ML. The predation activity of EMM111 towards MRSA was studied in more detail by monitoring the increase and decrease of fatty acid biomarkers characteristic of the predator and the prey, respectively [50]. EMM194 showed a significant antibacterial activity against Gram-negative bacteria (EC), and EMM183 showed a significant antagonistic activity against SA and MRSA. However, the most promising isolate against MRSA was EMM112, whereas the commercial antibiotics ceftriaxone (30 μg) and cefoxitin (30 μg) had no inhibitory impact on this multidrug-resistant bacteria. EMM112 also exhibited the highest antibacterial activity against both Gram-positive and Gram-negative negative bacteria and showed the largest inhibition zones against *M. luteus*.

### 2.6. Morphological, Physiological, and Molecular Characterization of the Selected Facultative Predator Actinobacteria Isolates

Morphological observation revealed that the four predatory Actinobacteria isolates were different (Table 3). The color of aerial and substrate mycelium of the strains varied. EMM111 produced a variety of pigments according to the medium composition, while the other isolates were not pigmented, except EMM183, which produced a brown pigment in ISP2. None of the four isolates produced melanin on peptone yeast extract agar (ISP 6). On the basis of the sequence of their 16S RNA, the four selected isolates were shown to belong to the genus *Streptomyces* (Table 4). The level of identity of EMM112 and EMM 183 with *Streptomyces coelicoflavus* and *Streptomyces mutabilis*, respectively, was 100%, whereas that of EMM111 and EMM194 with *Streptomyces griseoflavus* and *Streptomyces champavatii*, respectively, was 99%.

## 3. Discussion

Our study constitutes the first report confirming that oligotrophic environments like marine water might be a promising source of predatory Actinobacteria. Previous investigations demonstrated that in the absence of nutrients sources, diverse bacteria become predator and consume a variety of prey microorganisms as a nutritional resource [28,50,51,52]. Predatory bacteria are found in a wide variety of habitats, including seawater [27,53,54], and were reported to produce important therapeutic molecules active against human, animal, or plant pathogenic bacteria [55,56,57,58].

In the course of our study, we selected four different predatory Actinobacteria from Moroccan marine water (Figure 1 and Table 1). Our results demonstrated that predatory Actinobacteria could use Gram-positive and Gram-negative bacteria as feeding resources. They were all identified as belonging to the *Streptomyces* genus. Our results are consistent with previous reports showing that *Streptomyces* spp. are non-obligatory predators [33,42,59]. To date, only a few *Streptomyces* spp. predators of bacteria have been described. The first reported predatory Actinobacteria was a *Streptomyces* [41]. Then, the predation of *M. luteus* by *Streptoverticillium* was proposed [60]. Next, Kumbhar and coworkers [42] showed that *Streptomyces* are non-obligate epibiotic predators of a variety of microorganisms like *S. aureus*, *E. coli*, *Bacillus* spp., *Pseudomonas aeruginosa*, and *Klebsiella* spp. Recently, a marine *S. atrovirens* demonstrated a good predatory activity on a range of preys [33]. Finally, our study confirmed the existence of predatory activity in *Streptomyces* species. *S. griseoflavus* EMM111, *S. coelicoflavus* EMM112, and *S. mutabilis* EMM183 were already known as a terrestrial strain of *Streptomyces* [61,62,63], whereas *S. champavatii* EMM194 was previously isolated from a marine environment [64]. Some marine Actinobacteria can indeed have a terrestrial origin [65], being transported from a terrestrial to a marine environment by rivers, rainfall, wind, and spores leaching [65,66].

The results of the present study are potentially promising, since they indicate that predatory Actinobacteria can be easily isolated by a simple strategy requiring nutrient resources scarcity to induce the predatory behavior. In this study, marine water samples were simply plated on agar-agar plates by three methods. The first method used marine water dilution and direct spread of the prey directly onto the surface of the agar-agar plates. In the second method, the prey cells and molten agar-agar at 45 °C were shaken on sterile Scott bottles and poured into Petri dishes. Then, marine water dilutions were spread directly onto the surface. For the last technique, each marine water sample dilution and the prey were first transferred into sterile Petri dishes and mixed with molten agar-agar at 45 °C. In such condition, a great number of predatory bacteria could be isolated. The absence of growth of strains isolates on agar-agar control and their growth on agar-agar in the presence of *M. luteus* confirmed the predation. All four selected Actinobacteria isolates showed ability to grow on different types of bacterial cells (Gram-positive, Gram-negative, and multidrug-resistant bacteria), demonstrating the lack of specificity of the predation process that requires the excretion of lytic enzymes, in line with previous reports for other predatory bacteria [44]. Most of these species also produced anti-microbial molecules on Bennett medium. In condition of predation, these strains reduced the viability of *M. luteus*, but whether this is due to the predatory/lytic effect of these strains or to their ability to produce killing molecules is not known. Indeed, for instance, *S. champavatii* EMM194, which did not produce any molecule inhibiting the growth of the tested bacteria (except *E. coli*) when grown on Bennett medium, was still able to use all the bacteria tested as a prey. This suggests that predation and antibiotic-producing activities might not be linked. However, we cannot exclude that EMM194 produces specific bio-active molecules exclusively in the presence of its preys. Indeed, the production of bio-active secondary metabolites is often linked to the composition of the growth media that, in our condition, was exclusively constituted of components of living cells; in addition, the co-cultivation of Actinobacteria with other bacteria is known to lead to the production of compounds that are not produced in monoculture [22,23,24].

Since the four actinobacterial strains isolated were able to grow in the absence of prey on a variety of rich media, they should be considered as non-obligate predators. These bacteria are also able to produce anti-bacterial molecules, but the connection of these productions with the predatory process remains to be elucidated. The purification and elucidation of the structure of the antibacterial compounds produced by the selected isolates during the predation process or in monocultures are in progress.

## 4. Conclusions

This study constitutes the first report of the isolation predatory Actinobacteria from Moroccan marine water. The obtained data suggest that this oligotrophic environment is a promising reservoir of facultative predatory Actinobacteria. Four different facultative predatory Actinobacteria were isolated and identified. The four selected Actinobacteria showed a strong predatory activity spectrum and produced antibacterial substances on Bennett medium. It is possible that the antibacterial activity detected contributed to the killing and lysis of the bacteria, even if this could not be demonstrated firmly. Genome sequencing of the four predatory Actinobacteria is a crucial step to identify genes expressed during predation and specifically those directing the biosynthesis of potential killing compounds.

## 5. Materials and Methods 

### 5.1. Samples and Microbial Strains

#### 5.1.1. Sample Collection

Marine water samples were collected from nine different Moroccan locations of the Atlantic Ocean in April 2016. Sterile bottles were used to collect marine water samples. pH, temperature, and conductivity of the samples were measured at the moment of sampling, using a hand-held multiparameter device HI98194, Germany. The collected samples were transferred into sterile polythene bottles and stored at 4 °C for 24 h before further study.

#### 5.1.2. Bacterial Strains 

Prey cell strains used during investigations were *M. luteus* (ML) (ATCC381), *S. aureus* (SA) (ATCC 25923), methicillin-resistant *S. aureus* (MRSA) (NCTC 12493), *E. coli* (EC) (ATCC 8739), and ampicillin-resistant *E. coli* (AREC) (ATCC 35218). Bacterial inocula were prepared by growing cells on nutrient agar plates (3 g beef extract; 5 g peptone; 8 g sodium chloride; 15 g agar; 1000 mL distilled water; final pH 6.8 +/− 0.2) for 24 h at 37 °C.

### 5.2. Preparation of the Prey Cells and Screening of Predatory Actinobacteria Potential

We designed a new technique for the primary screening of predatory Actinobacteria isolates using *M. luteus* as a prey. The latter was cultivated on nutrient plates for 24 h at 37 °C, then collected, centrifuged at 4000 rpm for 10 min at 10 °C, and washed three times using sterile physiological water (NaCl 9 g/L). The final prey cell suspension was at a concentration of 10^12^ CFU/mL. Isolation of predatory Actinobacteria was performed from all water samples diluted up to 10^−6^ by three different methods. In the first method, 1 mL of *M. luteus* at the concentration 10^12^ CFU/mL and 100 µL of marine water dilution were plated directly on the surface of 20 mL of agar-agar plates containing 15 g of agar-agar per liter of sterile water. In the second method, 1 mL of *M. luteus* at the concentration 10^12^ CFU/mL was added to 20 mL of molten agar-agar at 45 °C, homogenized, and poured into Petri dishes, and 100 µL of marine water dilutions was spread directly onto the surface of the plates. In the last technique, 1 mL of each marine water sample dilution and M. *luteus* at the concentration 10^12^ CFU/mL was transferred into empty sterile Petri dishes, and 20 mL of molten agar-agar (45 °C) was poured on this mixture. The plates were incubated at 37 °C for 21 days. Each sample was analyzed separately and in triplicates, and several control experiments were carried out. The first control consisted of plates of agar-agar to test sterility. For the second control, 100 µL of water sample dilutions were plated on agar-agar. In the last control, agar-agar was inoculated with 1 mL of *M. luteus* washed live cells and incubated for 24 h at 37 °C. Since *M. luteus* could not grow on this medium, the plates were then flooded with 1 mL of nutrient broth to assess viability and abundance of the prey.

After 21 days of incubation, the growth of predatory Actinobacteria isolates was evaluated. Predatory Actinobacteria colonies were recognized on the basis of their morphological characteristics using light microscopy (G X10). The colony-forming units surrounded by zones of hydrolysis were selected as potential predators and purified. Most Actinobacteria showed vegetative and aerial mycelium, while others showed only substrate mycelium. Isolates were maintained on nutrient agar medium and stored at +4 °C for 2 months. Alternatively, cultures were re-suspended in 20% sterile glycerol and frozen at −80 °C.

### 5.3. Selection of Actinobacteria with Predatory Ability and Assessement of the Putative Prey Specificity of the Selected Actinobacteria Predators

The selection and the assessment of the putative prey specificity of t the selected purified isolates was tested using washed cell of *M. luteus*, *S. aureus*, *E.a coli*, methicillin-resistant *S. aureus*, and ampicillin-resistant *E. coli*. In this experiment, 1 mL of prey cells at 10^12^ CFU/mL were first plated on agar-agar plates, then 10 µL of predatory Actinobacteria isolates at 10^6^ CFU/mL was inoculated in the center of the plates.

### 5.4. Impact of Actinobacteria on M. Luteus Viability

We investigated the impact of Actinobacteria on *M. luteus* viability. For this, 1 mL of washed prey cells of *Mi. luteus* at 10^12^ CFU/mL was spread on agar-agar plates. Then, 10 µL of predatory Actinobacteria isolate at 10^6^ CFU/mL was inoculated in the center. In order to assess the prey viability at various distances of the Actinobacteria predator, samples taken at various distances were sub-cultured on nutrient agar plates for 24 h and 48 h at 37 °C. Control subcultures of the prey were also incubated in the absence of the predator.

### 5.5. Production of Antimicrobial Compounds by Actinobacteria Grown on Bennett Medium

The antibacterial activity was tested by the plate-diffusion method [67]. The four selected predatory Actinobacteria were grown on Bennett agar medium at 28 °C for 21 day. Agar cylinders (6 mm in diameter) were cut and placed on Mueller–Hinton agar, previously seeded by the test microorganisms. The plates were kept during 4 h at 4 °C for a good diffusion of the secondary metabolites produced, then incubated at 37 °C. Inhibition zones were determined after 24 hours. Antibacterial activity was evaluated in vitro using the five strains (ML, SA, EC, MRSA, and AREC) mentioned above. Antibiotics such as ceftriaxone (30 μg) and cefoxitin (30 μg) were used to reveal MDR bacteria according to Clinical and Laboratory Standards Institute guideline [68]. All tests were carried out in triplicate.

### 5.6. Statistical Analysis

The data obtained from the antibacterial activity were subjected to two-way analysis of variance (ANOVA). Significance of the differences between group means was assessed with the Tukey test; *p* < 0.05 was considered statistically significant. The statistical analysis was carried out using the software XLSTAT Version 2016.02.27444.

### 5.7. Morphological, Physiological, and Molecular Characterization of the Selected Facultative Predator Actinobacteria Isolates

The cultural, morphological, and physiological characterizations of the selected predatory Actinobacteria isolates were carried out according to the ISP [49]. For the molecular identification, the DNA of selected Actinobacteria was sequenced as described by Hopwood et al. 1985 [69]. The 16S rDNA was amplified by PCR with Taq DNA polymerase and primers 27F (5-AGAGTTTGATCCTGGCTCAG-3) and 1492R (5-TACGGYTACCTTGTTACGACTT-3). The conditions for thermal cycling were as follows: denaturation of the target DNA at 98 °C for 3 min followed by 30 cycles at 94 °C for 1 min, primer annealing at 52 °C for 1 min, and primer extension at 72 °C for 5 min. At the end of the cycling, the reaction mixture was held at 72 °C for 5 min and then cooled to 4 °C. The PCR product obtained was sequenced using an automated sequencer and the same primers used for sequence determination (Macrogen Inc., Seoul, Korea). The sequence was compared for similarity with the reference sequences of bacteria species contained in genomic database banks, using the NCBI Blast available at http://www.ncbi.nlm.nih.gov/

## Figures and Tables

**Table 1:** Origin and number of predatory Actinobacteria isolates.

Samples	Number of Predatory Bacteria	Number of Predatory Actinobacteria	Method of Isolation	Code
**1**	23	2	2	EMM111
			1	EMM112
**2**	12	1	1	EMM111
**3**	17	1	1	EMM111
**4**	6	1	1	EMM111
**5**	11	0	-	-
**6**	5	1	1	EMM111
**7**	18	1	1	EMM111
**8**	30	1	2	EMM183
**9**	20	1	2	EMM194

**Table 2:** Comparison of the predatory activity and the antibacterial activity on rich medium of four predatory Actinobacteria isolates.

Strains	Predatory Activity					Antibacterial Activity in a Rich Medium (Bennett)				
	ML	SA	EC	MRSA	AREC	ML	SA	EC	MRSA	AREC
**EMM111**	+	+	+	+	+	22.16 ± 0.7	17.5 ± 0.5	0	16.3	0
**EMM112**	+	+	+	+	+	45 ± 1	35.4 ± 0.9	23 ± 1	34.6 ± 0.5	15 ± 1
**EMM183**	+	+	+	+	+	0	35 ± 1	0	23.26 ± 3.2	0
**EMM194**	+	+	+	+	+	0	0	4.4 ± 0.5	0	0

Predatory activity is expressed by the presence (+) or absence (0). The experiments were repeated three times (n = 3) with each independent assay. Antibacterial activity in a rich medium (Bennett) is expressed by inhibition diameter (mm). Each value represents the mean ± SD of three replicates.

**Table 3:** Morphological and physiological characteristics of four selected predatory Actinobacteria isolates.

Properties	EMM111	EMM112	EMM183	EMM194
**ISP2**				
**Color of aerial mycelium**	beige	orange	white	white
**Color of substrate mycelium**	white	brown	beige	white
**Diffusible pigment produced**	orange	-	brown	-
**ISP6**				
**Color of aerial mycelium**	beige	grey	white	white
**Color of substrate mycelium**	beige	black	white	white
**Diffusible pigment produced**	-	-	-	-
**ISP7**				
**Color of aerial mycelium**	beige	grey	pink	white
**Color of substrate mycelium**	white	black	pink	brown
**Diffusible pigment produced**	grey	-	-	-

The sign “-” means negative.

**Table 4:** Comparison of percent similarities between our 16S rRNA gene sequence and sequences present in the genomic database banks using NCBI BLAST.

Strains	Percentage of Sequence Identity (%)	Actinobacteria Strains	Accession
**EMM111**	99	*Streptomyces griseoflavus*	NR_042291.1
**EMM112**	100	*Streptomyces coelicoflavus*	NR_041175.1
**EMM183**	100	*Streptomyces mutabilis*	NR_044139.1
**EMM194**	99	*Streptomyces champavatii*	NR_115669.1

## Supplementary Materials

The following are available online at https://www.mdpi.com/2079-6382/9/2/91/s1, Table S1: Sampling sites and some physicochemical parameters.
