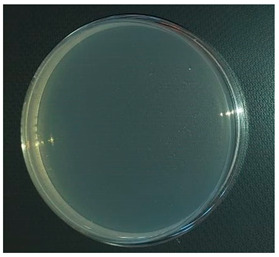# Correction: Ibrahimi et al. Marine Actinobacteria: Screening for Predation Leads to the Discovery of Potential New Drugs against Multidrug-Resistant Bacteria. *Antibiotics* 2020, *9*, 91

**DOI:** 10.3390/antibiotics13040288

**Published:** 2024-03-22

**Authors:** Manar Ibrahimi, Wassila Korichi, Mohamed Hafidi, Laurent Lemee, Yedir Ouhdouch, Souad Loqman

**Affiliations:** 1 Laboratory of Microbial Biotechnologies, Agrosciences and Environment (BioMAgE), Faculty of Sciences Semlalia, Cadi Ayyad University, PO Box 2390, Marrakesh, Morocco; wassila.korichi@edu.uca.ac.ma (W.K.); hafidi@uca.ma (M.H.); ouhdouch@uca.ac.ma (Y.O.); 2 Institut de Chimie des Milieux et Matériaux de Poitiers (IC2MP - CNRS UMR 7285), Université de Poitiers, 4 rue Michel Brunet – TSA 51106, 86073 Poitiers Cedex 9, France; laurent.lemee@univ-poitiers.fr; 3 Laboratory of Microbiology and Virology, Faculty of Medicine and Pharmacy, Cadi Ayyad University, PO Box 7010, Marrakesh, Morocco; s.loqman@uca.ma; 4 Agro Bio Sciences Program, Mohammed VI Polytechnic University (UM6P), Benguerir, 43150, Morocco

## Error in Figure

In the original publication, there was a mistake in Figure 1b as published [1]. Figure 1b duplicates Figure 1a. The corrected Figure 1b appears below. The authors state that the scientific conclusions are unaffected. This correction was approved by the Academic Editor. The original publication has also been updated.